# Supplementary material for: Inhaled Nitric Oxide for Clinical Management of COVID-19: A Systematic Review and Meta-Analysis
Source: Int J Environ Res Public Health. 2022 Oct 6;19(19):12803. doi: 10.3390/ijerph191912803 (PMC9566710; doi:10.3390/ijerph191912803)
Supplement: Supplementary file 1 [file ijerph-19-12803-s001.zip › ijerph-1905034-supplementary.pdf]

Table S1. Quality assessment using modified Newcastle-Ottawa Scale.

	SELECTION		COMPARABILITY	OUTCOME		OVERALL SCORE
**Author, years**	**Adequate sample size (≥10 patients).**	**Adequate diagnosis (PaO2/FiO2 < 150 mmHg)**	**Result controlled for contributory factors**	**Response adequately described (improved PaO2/FiO2)**	**Patients followed up during and after treatment**	**Score >3 stars = Low bias risk**
Abman et al, 2022 (1)	*	*		*	*	****
Abou-Arab et al, 2020 (2)	*	*		*	*	****
Bagate et al, 2020 (3)	*	*	*	*	*	*****
Bonizzoli et al, 2022 (4)	*	*		*	*	****
Caplan et al, 2021 (5)	*	*		*	*	****
Chandel et al, 2021 (6)	*		*		*	***
DeGrado et al, 2020 (7)	*	*		*	*	****
Fakhr et al, 2021 (8)	*	*			*	***
Ferrari et al, 2020 (9)	*	*		*	*	****
Herranz et al, 2021 (10)	*	*		*	*	****
Laghlam et al, 2021 (11)	*	*	*	*	*	*****
Lubinsky et al, 2022 (12)	*	*		*	*	****
Matthews et al, 2022 (13)	*	*		*	*	****

Parikh et al, 2020 (14)	*			*	*	***
Robba et al, 2021 (15)	*	*	*	*	*	*****
Tavazzi et al, 2020 (16)	*	*		*	*	****
Ziehr et al, 2021 (17)	*	*		*	*	****

Database: Embase Classic+Embase <1947 to 2022 May 18>
Search Strategy:
----
1 Nitric Oxide.mp. or exp nitric oxide/ (277326)
2 nitrogen monoxide.mp. (879)
3 mononitrogen monoxide.mp. (0)
4 1 or 2 or 3 (277487)
5 SARS-CoV-2.mp. or exp Severe acute respiratory syndrome coronavirus 2/ (112816)
6 2019 Novel Coronavirus*.mp. [mp=title, abstract, heading word, drug trade name, original title, device manufacturer, drug manufacturer, device trade name, keyword heading word, floating subheading word, candidate term word] (1796)
7 2019-nCoV*.mp. [mp=title, abstract, heading word, drug trade name, original title, device manufacturer, drug manufacturer, device trade name, keyword heading word, floating subheading word, candidate term word] (2271)
8 COVID-19*.mp. [mp=title, abstract, heading word, drug trade name, original title, device manufacturer, drug manufacturer, device trade name, keyword heading word, floating subheading word, candidate term word] (239498)
9 Coronavirus Disease 2019*.mp. [mp=title, abstract, heading word, drug trade name, original title, device manufacturer, drug manufacturer, device trade name, keyword heading word, floating subheading word, candidate term word] (223189)
10 Coronavirus Disease-19*.mp. [mp=title, abstract, heading word, drug trade name, original title, device manufacturer, drug manufacturer, device trade name, keyword heading word, floating subheading word, candidate term word] (2592)
11 SARS Coronavirus 2*.mp. [mp=title, abstract, heading word, drug trade name, original title, device manufacturer, drug manufacturer, device trade name, keyword heading word, floating subheading word, candidate term word] (4573)
12 SARS-CoV-2*.mp. [mp=title, abstract, heading word, drug trade name, original title, device manufacturer, drug manufacturer, device trade name, keyword heading word, floating subheading word, candidate term word] (97920)
13 Severe Acute Respiratory Syndrome Coronavirus 2*.mp. [mp=title, abstract, heading word, drug trade name, original title, device manufacturer, drug manufacturer, device trade name, keyword heading word, floating subheading word, candidate term word] (74484)
14 Wuhan Coronavirus*.mp. [mp=title, abstract, heading word, drug trade name, original title, device manufacturer, drug manufacturer, device trade name, keyword heading word, floating subheading word, candidate term word] (48)
15 Wuhan Seafood Market Pneumonia Virus*.mp. [mp=title, abstract, heading word, drug trade name, original title, device manufacturer, drug manufacturer, device trade name, keyword heading word, floating subheading word, candidate term word] (4)
16 5 or 6 or 7 or 8 or 9 or 10 or 11 or 12 or 13 or 14 or 15 (282662)
17 4 and 16 (1056)
18 limit 17 to english language (1044)
19 limit 18 to conference abstract status (98)
20 18 not 19 (946)
21 limit 20 to "systematic review" (53)
22 20 not 21 (893)
23 limit 22 to "reviews (best balance of sensitivity and specificity)" (285)
24 22 not 23 (608)
25 limit 24 to letter (101)
26 24 not 25 (507)
27 limit 26 to editorial (39)
28 26 not 27 (468)
29 limit 28 to conference paper (1)
30 28 not 29 (467)
31 limit 30 to chapter (3)
32 30 not 31 (464)
33 limit 32 to human (234)
****

Database: Ovid MEDLINE(R) ALL <1946 to May 18, 2022>
Search Strategy:
----
1 Nitric Oxide.mp. or exp Nitric Oxide/ (185457)
2 nitrogen monoxide.mp. (609)
3 mononitrogen monoxide.mp. (1)
4 1 or 2 or 3 (185696)
5 SARS-CoV-2.mp. or exp SARS-CoV-2/ (161679)
6 2019 Novel Coronavirus*.mp. [mp=title, abstract, original title, name of substance word, subject heading word, floating sub-heading word, keyword heading word, organism supplementary concept word, protocol supplementary concept word, rare disease supplementary concept word, unique identifier, synonyms] (1702)
7 2019-nCoV*.mp. [mp=title, abstract, original title, name of substance word, subject heading word, floating sub-heading word, keyword heading word, organism supplementary concept word, protocol supplementary concept word, rare disease supplementary concept word, unique identifier, synonyms] (2409)
8 COVID-19*.mp. [mp=title, abstract, original title, name of substance word, subject heading word, floating sub-heading word, keyword heading word, organism supplementary concept word, protocol supplementary concept word, rare disease supplementary concept word, unique identifier, synonyms] (246571)
9 Coronavirus Disease 2019.mp. [mp=title, abstract, original title, name of substance word, subject heading word, floating sub-heading word, keyword heading word, organism supplementary concept word, protocol supplementary concept word, rare disease supplementary concept word, unique identifier, synonyms] (43317)
10 Coronavirus Disease-19.mp. [mp=title, abstract, original title, name of substance word, subject heading word, floating sub-heading word, keyword heading word, organism supplementary concept word, protocol supplementary concept word, rare disease supplementary concept word, unique identifier, synonyms] (2288)
11 SARS Coronavirus 2*.mp. [mp=title, abstract, original title, name of substance word, subject heading word, floating sub-heading word, keyword heading word, organism supplementary concept word, protocol supplementary concept word, rare disease supplementary concept word, unique identifier, synonyms] (383)
12 SARS-CoV-2*.mp. [mp=title, abstract, original title, name of substance word, subject heading word, floating sub-heading word, keyword heading word, organism supplementary concept word, protocol supplementary concept word, rare disease supplementary concept word, unique identifier, synonyms] (161696)
13 Severe Acute Respiratory Syndrome Coronavirus 2*.mp. [mp=title, abstract, original title, name of substance word, subject heading word, floating sub-heading word, keyword heading word, organism supplementary concept word, protocol supplementary concept word, rare disease supplementary concept word, unique identifier, synonyms] (24745)
14 Wuhan Coronavirus*.mp. [mp=title, abstract, original title, name of substance word, subject heading word, floating sub-heading word, keyword heading word, organism supplementary concept word, protocol supplementary concept word, rare disease supplementary concept word, unique identifier, synonyms] (43)
15 Wuhan Seafood Market Pneumonia Virus*.mp. [mp=title, abstract, original title, name of substance word, subject heading word, floating sub-heading word, keyword heading word, organism supplementary concept word, protocol supplementary concept word, rare disease supplementary concept word, unique identifier, synonyms] (4)
16 5 or 6 or 7 or 8 or 9 or 10 or 11 or 12 or 13 or 14 or 15 (254518)
17 4 and 16 (347)
18 limit 17 to english language (278)
****

Figure S1. Search Strategy for Medline and Embase Databases.

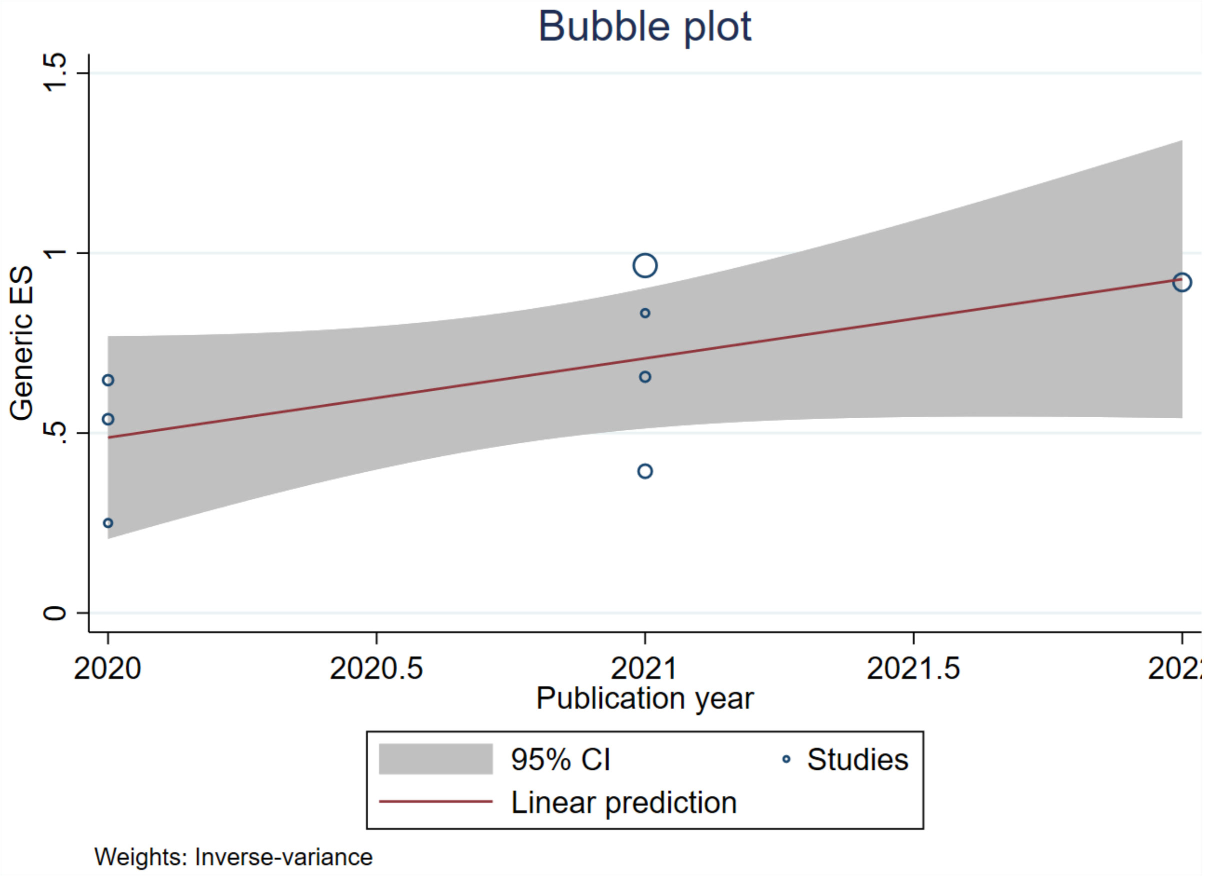

Figure S2. Bubble plot for meta-regression analysis of the effect of publication year on the response to inhaled nitric oxide in patients with COVID-19-related acute respiratory response syndrome.

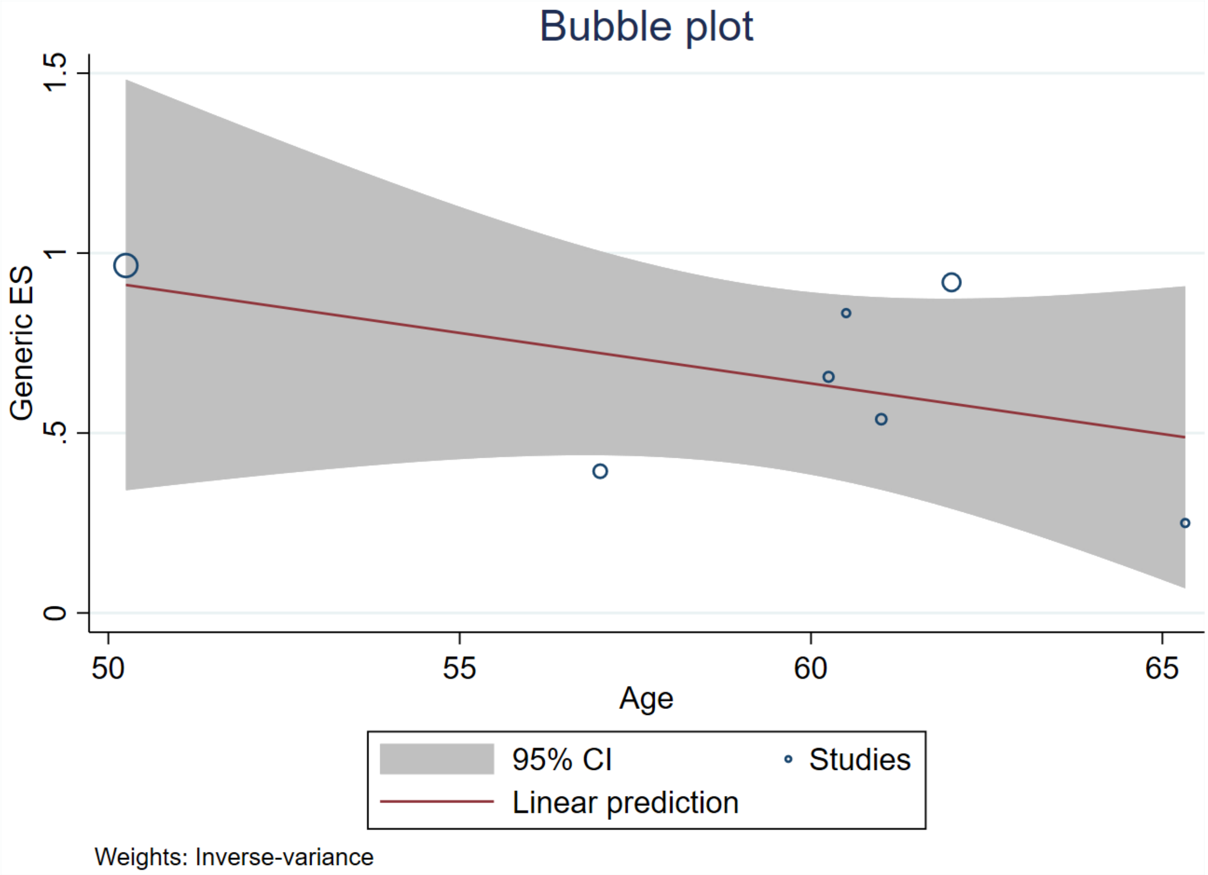

Figure S3. Bubble plot for meta-regression analysis of the effect of mean reported age on the response to inhaled nitric oxide in patients with COVID-19-related acute respiratory response syndrome.

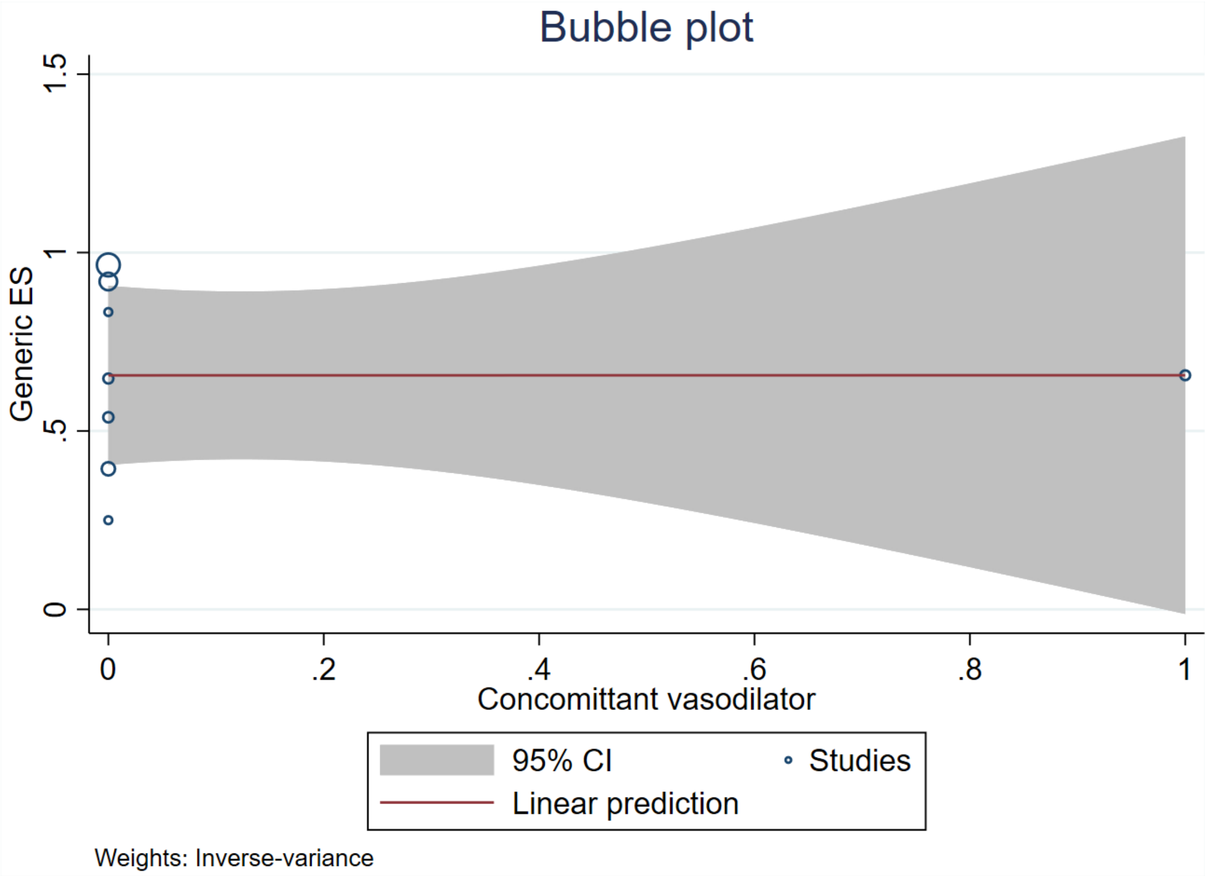

Figure S4. Bubble plot for meta-regression analysis of the effect of concomitant vasodilator use on the response to inhaled nitric oxide in patients with COVID-19-related acute respiratory response syndrome.

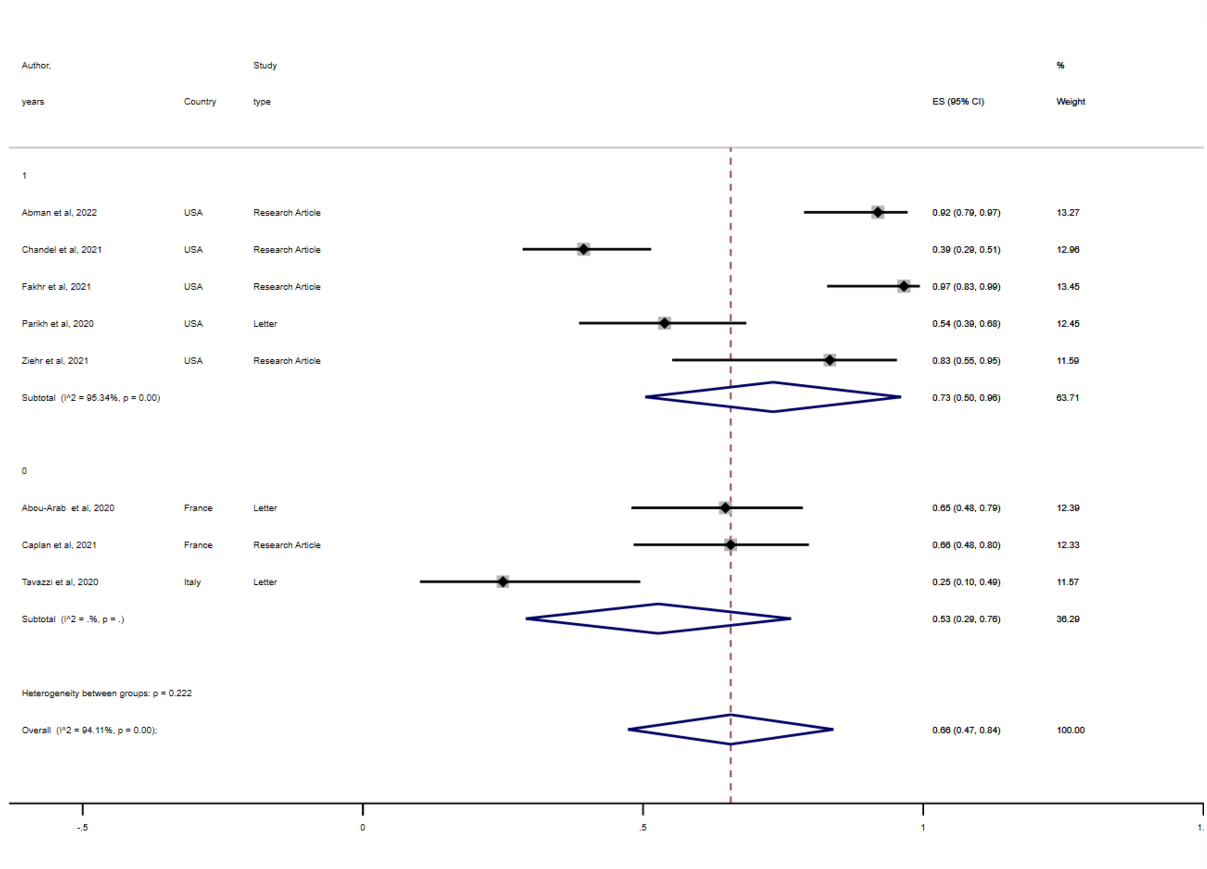

Figure S5. *Pooled prevalence of rate of response to inhaled nitric oxide (iNO) in patients with COVID-19 grouped by whether studies were conducted in United State of America, USA (1) or not (0). Red dotted line represents the overall response rate (0.66; USA = 0.73; Others = 0.53). Lateral edges of the blue diamond represent the extreme/limits of the 95% confidence interval (95% CI: 0.47, 0.84; USA = 50, 96; Others = 29, 76). ES = Effect size, USA = United States of America.*

References:

1. Abman SH, Fox NR, Malik MI, Kelkar SS, Corman SL, Rege S, et al. Real-world use of inhaled nitric oxide therapy in patients with COVID-19 and mild-to-moderate acute respiratory distress syndrome. Drugs Context. 2022;11.
2. Abou-Arab O, Huette P, Debouvries F, Dupont H, Jounieaux V, Mahjoub Y. Inhaled nitric oxide for critically ill Covid-19 patients: a prospective study. Critical Care. 2020;24(1) (no pagination).
3. Bagate F, Tuffet S, Masi P, Perier F, Razazi K, de Prost N, et al. Rescue therapy with inhaled nitric oxide and almitrine in COVID-19 patients with severe acute respiratory distress syndrome. Annals of Intensive Care. 2020;10(1) (no pagination).
4. Bonizzoli M, Lazzeri C, Cianchi G, Guetti C, Fulceri GE, Socci F, et al. Effects of rescue inhaled nitric oxide on right ventricle and pulmonary circulation in severe COVID-related acute respiratory distress syndrome. Journal of Critical Care. 2022;(no pagination).
5. Caplan M, Goutay J, Bignon A, Jaillette E, Favory R, Mathieu D, et al. Almitrine Infusion in Severe Acute Respiratory Syndrome Coronavirus 2-Induced Acute Respiratory Distress Syndrome: A Single-Center Observational Study. Critical Care Medicine. 2021;49(2):e191-e8.
6. Chandel A, Patolia S, Ahmad K, Aryal S, Brown AW, Sahjwani D, et al. Inhaled Nitric Oxide via High-Flow Nasal Cannula in Patients with Acute Respiratory Failure Related to COVID-19. Clinical Medicine Insights: Circulatory, Respiratory and Pulmonary Medicine. 2021;15(no pagination).
7. DeGrado JR, Szumita PM, Schuler BR, Dube KM, Lenox J, Kim EY, et al. Evaluation of the Efficacy and Safety of Inhaled Epoprostenol and Inhaled Nitric Oxide for Refractory Hypoxemia in Patients With Coronavirus Disease 2019. Crit. 2020;2(10):e0259.
8. Safaee Fakhr B, Di Fenza R, Gianni S, Wiegand SB, Miyazaki Y, Araujo Morais CC, et al. Inhaled high dose nitric oxide is a safe and effective respiratory treatment in spontaneous breathing hospitalized patients with COVID-19 pneumonia. Nitric Oxide - Biology and Chemistry. 2021;116:7-13.
9. Ferrari M, Santini A, Protti A, Andreis DT, Iapichino G, Castellani G, et al. Inhaled nitric oxide in mechanically ventilated patients with COVID-19. Journal of Critical Care. 2020;60:159-60.
10. Herranz L, da Silveira JG, Trocado LFL, Alvaraes AL, Fittipaldi J. Inhaled Nitric Oxide in Patients with Severe COVID-19 Infection at Intensive Care Unit - A Cross Sectional Study. J. 2021;7(4):318-9.
11. Laghlam D, Rahoual G, Malvy J, Estagnasie P, Brusset A, Squara P. Use of Almitrine and Inhaled Nitric Oxide in ARDS Due to COVID-19. Frontiers in Medicine. 2021;8 (no pagination).
12. Lubinsky AS, Brosnahan SB, Lehr A, Elnadoury O, Hagedorn J, Garimella B, et al. Inhaled pulmonary vasodilators are not associated with improved gas exchange in mechanically ventilated patients with COVID-19: A retrospective cohort study. Journal of Critical Care. 2022;69 (no pagination).
13. Matthews L, Baker L, Ferrari M, Sanchez W, Pappachan J, Grocott MP, et al. Compassionate use of Pulmonary Vasodilators in Acute Severe Hypoxic Respiratory Failure due to COVID-19. Journal of intensive care medicine. 2022:8850666221086521.
14. Parikh R, Wilson C, Weinberg J, Gavin D, Murphy J, Reardon CC. Inhaled nitric oxide treatment in spontaneously breathing COVID-19 patients. Therapeutic Advances in Respiratory Disease. 2020;14:1753466620933510.
15. Robba C, Ball L, Battaglini D, Cardim D, Moncalvo E, Brunetti I, et al. Early effects of ventilatory rescue therapies on systemic and cerebral oxygenation in mechanically ventilated COVID-19 patients with acute respiratory distress syndrome: a prospective observational study. Critical Care. 2021;25(1) (no pagination).
16. Tavazzi G, Pozzi M, Mongodi S, Dammassa V, Romito G, Mojoli F. Correction to: Inhaled nitric oxide in patients admitted to intensive care unit with COVID-19 pneumonia. Crit Care. 2020;24(1):665.
17. Ziehr DR, Alladina J, Wolf ME, Brait KL, Malhotra A, La Vita C, et al. Respiratory Physiology of Prone Positioning With and Without Inhaled Nitric Oxide Across the Coronavirus Disease 2019 Acute Respiratory Distress Syndrome Severity Spectrum. Crit. 2021;3(6):e0471.
